# Telemedizin in der Schlaganfallversorgung – versorgungsrelevant für Deutschland

**DOI:** 10.1007/s00115-021-01137-6

**Published:** 2021-05-27

**Authors:** J. Barlinn, S. Winzer, H. Worthmann, C. Urbanek, K. G. Häusler, A. Günther, H. Erdur, M. Görtler, L. Busetto, C. Wojciechowski, J. Schmitt, Y. Shah, B. Büchele, P. Sokolowski, T. Kraya, S. Merkelbach, B. Rosengarten, K. Stangenberg-Gliss, J. Weber, F. Schlachetzki, M. Abu-Mugheisib, M. Petersen, A. Schwartz, F. Palm, A. Jowaed, B. Volbers, P. Zickler, J. Remi, J. Bardutzky, J. Bösel, H. J. Audebert, G. J. Hubert, C. Gumbinger

**Affiliations:** 1 Klinik für Neurologie, Universitätsklinikum Dresden, Fetscherstraße 74, 01307 Dresden, Deutschland; 2 Klinik für Neurologie, Medizinische Hochschule Hannover, Hannover, Deutschland; 3 Klinik für Neurologie, Klinikum der Stadt Ludwigshafen, Ludwigshafen, Deutschland; 4 Neurologische Klinik und Poliklinik, Universitätsklinikum Würzburg, Würzburg, Deutschland; 5 Klinik für Neurologie, Universitätsklinikum Jena, Jena, Deutschland; 6 Klinik und Hochschulambulanz für Neurologie, Charité – Universitätsmedizin Berlin, Berlin, Deutschland; 7 Klinik für Neurologie, Universitätsklinikum Magdeburg, Magdeburg, Deutschland; 8 Klinik für Neurologie, Universitätsklinikum Heidelberg, Heidelberg, Deutschland; 9 Zentrum für Evidenzbasierte Gesundheitsversorgung, Universitätsklinikum Dresden, Dresden, Deutschland; 10 Klinik für Neurologie, Klinikum Kassel, Kassel, Deutschland; 11 Klinik für Neurologie, Städtisches Klinikum Karlsruhe, Karlsruhe, Deutschland; 12 Klinik für Neurologie und neurologische Intensivmedizin, Fachkrankenhaus Hubertusburg, Hubertusburg, Deutschland; 13 Klinik für Neurologie, Klinikum St.Georg Leipzig, Leipzig, Deutschland; 14 Klinik für Neurologie, Heinrich-Braun-Klinikum Zwickau, Zwickau, Deutschland; 15 Klinik für Neurologie, Klinikum Chemnitz, Chemnitz, Deutschland; 16 Klinik für Neurologie, BG Klinikum Unfallkrankenhaus Berlin, Berlin, Deutschland; 17 Klinik für Neurologie, Universität Regensburg, Regensburg, Deutschland; 18 Klinik für Neurologie, Städtisches Klinikum Braunschweig, Braunschweig, Deutschland; 19 Klinik für Neurologie, Klinikum Osnabrück, Osnabrück, Deutschland; 20 Klinik für Neurologie, Klinikum Region Hannover, Hannover, Deutschland; 21 Klinik für Neurologie, Helios Klinikum Schleswig, Schleswig, Deutschland; 22 Klinik für Neurologie, Westküstenkliniken Heide, Heide, Deutschland; 23 Klinik für Neurologie, Universitätsklinikum Erlangen, Erlangen, Deutschland; 24 Klinik für Neurologie und Klinische Neurophysiologie, Universitätsklinikum Augsburg, Augsburg, Deutschland; 25 Klinik für Neurologie, Klinikum der LMU München-Großhadern, München, Deutschland; 26 Klinik für Neurologie, Universitätsklinikum Freiburg, Freiburg, Deutschland; 27 Centrum für Schlaganfallforschung Berlin, Charité – Universitätsmedizin Berlin, Berlin, Deutschland; 28 Klinik für Neurologie, München-Klinik Harlaching, München, Deutschland

**Keywords:** Schlaganfall, Stroke-Unit, Telemedizin, Schlaganfall-Netzwerk, Umfragestudie, Stroke, Stroke unit, Telemedicine, Stroke networks, Survey

## Abstract

**Hintergrund und Ziel:**

Telemedizinische Schlaganfall-Netzwerke tragen dazu bei, die Schlaganfallversorgung und insbesondere den Zugang zu zeitkritischen Schlaganfalltherapien in vorrangig strukturschwachen, ländlichen Regionen zu gewährleisten. Ziel ist eine Darstellung der Nutzungsfrequenz und regionalen Verteilung dieser Versorgungsstruktur.

**Methoden:**

Die Kommission „Telemedizinische Schlaganfallversorgung“ der Deutschen Schlaganfall-Gesellschaft führte eine Umfragestudie in allen Schlaganfall-Netzwerken durch.

**Ergebnisse:**

In Deutschland sind 22 telemedizinische Schlaganfall-Netzwerke aktiv, welche insgesamt 43 Zentren (pro Netzwerk: Median 1,5, Interquartilsabstand [IQA] 1–3) sowie 225 Kooperationskliniken (pro Netzwerk: Median 9, IQA 4–17) umfassen und an einem unmittelbaren Zugang zur Schlaganfallversorgung für 48 Mio. Menschen teilhaben. Im Jahr 2018 wurden 38.211 Telekonsile (pro Netzwerk: Median 1340, IQA 319–2758) durchgeführt. Die Thrombolyserate betrug 14,1 % (95 %-Konfidenzintervall 13,6–14,7 %), eine Verlegung zur Thrombektomie wurde bei 7,9 % (95 %-Konfidenzintervall 7,5–8,4 %) der ischämischen Schlaganfallpatienten initiiert. Das Finanzierungssystem ist uneinheitlich mit einem Vergütungssystem für die Zentrumsleistungen in nur drei Bundesländern.

**Diskussion:**

Etwa jeder 10. Schlaganfallpatient wird telemedizinisch behandelt. Die telemedizinischen Schlaganfall-Netzwerke erreichen vergleichbar hohe Lyseraten und Verlegungen zur Thrombektomie wie neurologische Stroke-Units und tragen zur Sicherstellung einer flächendeckenden Schlaganfallversorgung bei. Eine netzwerkübergreifende Sicherstellung der Finanzierung und einheitliche Erhebung von Qualitätssicherungsdaten haben das Potenzial diese Versorgungsstruktur zukünftig weiter zu stärken.

**Zusatzmaterial online:**

Die Onlineversion dieses Beitrags (10.1007/s00115-021-01137-6) enthält weiteres Zusatzmaterial (Fragebogen). Beitrag und Zusatzmaterial stehen Ihnen auf www.springermedizin.de zur Verfügung. Bitte geben Sie dort den Beitragstitel in die Suche ein, das Zusatzmaterial finden Sie beim Beitrag unter „Ergänzende Inhalte“.

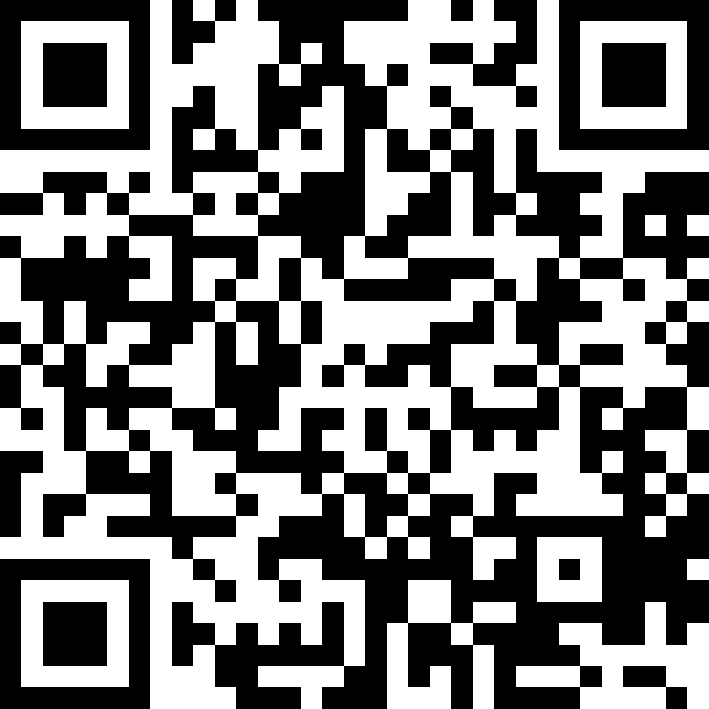

## Abstract (en)

**Background and objective:**

Telemedical stroke networks improve stroke care and provide access to time-dependent acute stroke treatment in predominantly rural regions. The aim is a presentation of data on its utility and regional distribution.

**Methods:**

The working group on telemedical stroke care of the German Stroke Society performed a survey study among all telestroke networks.

**Results:**

Currently, 22 telemedical stroke networks including 43 centers (per network: median 1.5, interquartile range, IQR, 1–3) as well as 225 cooperating hospitals (per network: median 9, IQR 4–17) operate in Germany and contribute to acute stroke care delivery to 48 million people. In 2018, 38,211 teleconsultations (per network: median 1340, IQR 319–2758) were performed. The thrombolysis rate was 14.1% (95% confidence interval 13.6–14.7%) and transfer for thrombectomy was initiated in 7.9% (95% confidence interval 7.5–8.4%) of ischemic stroke patients. Financial reimbursement differs regionally with compensation for telemedical stroke care in only three federal states.

**Conclusion:**

Telemedical stroke care is utilized in about 1 out of 10 stroke patients in Germany. Telemedical stroke networks achieve similar rates of thrombolysis and transfer for thrombectomy compared with neurological stroke units and contribute to stroke care in rural regions. Standardization of network structures, financial assurance and uniform quality measurements may further strengthen the importance of telestroke networks in the future.

Die telemedizinischen Schlaganfall-Netzwerke in Deutschland leisten einen wesentlichen Beitrag zur flächendeckenden Versorgung von Schlaganfallpatienten, indem Zentren den angebundenen Kooperationskliniken zeitkritische Behandlungsempfehlungen aussprechen und diese unterstützen, eine Schlaganfallexpertise vor Ort aufbauen. In dieser Arbeit werden erstmals bundesweit systematisch erfasste Daten zu Struktur und Leistungen der Netzwerke vorgestellt und eine Optimierung der Ressourcenallokation diskutiert.

## Hintergrund

Zur flächendeckenden Sicherstellung zeitkritischer Schlaganfalltherapien wurden seit Beginn der 2000er-Jahre telemedizinische Schlaganfall-Netzwerke etabliert [1], wobei Schlaganfallzentren dem Netzwerk zugehörige Kooperationskliniken beraten und Behandlungsempfehlungen aussprechen. Die telemedizinische Beurteilung von Schlaganfallpatienten und deren zerebraler Bildgebung ist zuverlässig und ermöglicht die sichere und effektive Durchführung der systemischen Lysetherapie [2–4]. Schlaganfall-Netzwerke erreichen dabei vergleichbare Lyseraten und Prozesszeiten wie nichttelemedizinisch operierende Schlaganfallzentren [5, 6]. Auch für die Rekanalisation mittels Thrombektomie, die seit 2015 als Goldstandard für die Behandlung von Großgefäßverschlüssen gilt und nur in ausgewiesenen Zentren zur Verfügung steht, kann die telemedizinische Patientenselektion zur Optimierung der Behandlungsraten beitragen [7, 8]. Dies ist vor dem Hintergrund weiter Entfernungen zu Zentren mit Thrombektomieversorgung wichtig [9].

Eine zentrale Herausforderung ist, gerade beim potenziell lebensbedrohlichen und zeitkritischen Schlaganfall die unmittelbare wohnortnahe Versorgung abzudecken und zugleich ausgewählten Patienten den Zugang zur Spezialversorgung in Schlaganfallzentren zu ermöglichen. Wir groß der Beitrag der Schlaganfall-Netzwerke zur Schlaganfallversorgung in Deutschland ist, war bislang unklar. Trotz zunehmender Bedeutung der Tele- und Netzwerkmedizin beim Schlaganfall fehlen bislang bundesweite Daten über Nutzungsfrequenz und regionale Verteilung dieser Versorgungsstruktur. Ziel dieser Arbeit ist die Erfassung der bestehenden Telemedizinstrukturen, die Darstellung ihres Beitrages zur Schlaganfallversorgung und die Identifizierung von Optimierungspotenzial.

## Methode

### Umfragestudie

Es erfolgte eine Umfragestudie in Form einer standardisierten Befragung in den an der Kommission „Telemedizinische Schlaganfallversorgung“ der Deutschen Schlaganfall-Gesellschaft beteiligten 22 telemedizinischen Schlaganfall-Netzwerken. Die Identifizierung der Netzwerke erfolgte im Vorfeld durch Kommunikation, Recherche und Abgleich mit Anbietern der Telemedizintechnik. Somit umfasste die systematische Stichprobe alle in Deutschland identifizierten aktiven Schlaganfall-Netzwerke. Die Fragebogenentwicklung erfolgte entsprechend einer Gliederung in die Bereiche Basisdaten/Struktur, Konsile/Leistungszahlen, Koordination/Netzwerkarbeit, Standards, Finanzierung und Technik, die in getrennten Frageblöcken abgebildet wurden. Für die einzelnen Aspekte wurden Fragen formuliert, die sowohl quantitative und qualitative Gesichtspunkte umfassten. Die Klassifikation der Fragen umfasste offene Fragen, Mehrfachantwortfragen und Hybridfragen. Die Erhebung der Leistungszahlen bezog sich auf das Jahr 2018, alle übrigen Punkte erfragten den Stand zum Zeitpunkt der Befragung (ab 01/2020). Zur Überprüfung der Funktion und Verständlichkeit des Fragebogens erfolgte ein Pretest in einer Stichprobe von 6 Netzwerken (01.12.2019), woraufhin Anpassungen an Frage- und Antwortformulierungen erfolgten. Maßnahmen zur Verringerung der Nonresponse umfassten die Ankündigung der Befragung sowie schriftliche und telefonische Kontaktaufnahmen. Der Schluss der Datenbank erfolgte am 30.07.2020.

### Statistik

In Abhängigkeit von Verteilung und Skalenniveau erfolgte die Darstellung kontinuierlicher Variablen als Median mit Angabe des Interquartilabstandes (IQA) oder als Mittelwert (MW) ± Standardabweichung (SD). Häufigkeiten wurden in Prozent und bei ausgewählten Variablen mit Angabe des 95 %-Wald-Konfidenzintervalls dargestellt. Für die deskriptive Analyse wurden ausschließlich verfügbare Werte im Sinne eines paarweisen Ausschlusses berücksichtigt.

Die statistische Analyse wurde mit dem Programm Stata 12.1 (StataCorp, College Station, Texas, USA) durchgeführt. Die kartografischen Darstellungen wurde mittels Geopandas GIS Version 0.7, Pandas Version 1.0.4 und Matplotlib Version 3.2.1 erstellt (Python 3.8.2). Die Einzugsgebietsanalysen, basierend auf Daten zur Bevölkerungsstatistik des Jahres 2011 des Statistischen Bundesamtes (destatis.de), wurden mithilfe der Software und Daten der Targomo GmbH (Potsdam, Deutschland) erstellt.

## Ergebnisse

### Umfragestudie

Der Rücklauf der Fragebögen war vollständig (22/22). Zur Vervollständigung einzelner Items in den Fragebögen erfolgten Kontaktaufnahmen mit 13 Netzwerken. Einzelne Items waren bei 7/22 (31,8 %) Netzwerken nicht verfügbar, hauptsächlich begründet durch ein fehlendes einheitliches Qualitätssicherungsverfahren.

### Strukturen der telemedizinischen Schlaganfall-Netzwerke

**Figure 1:**
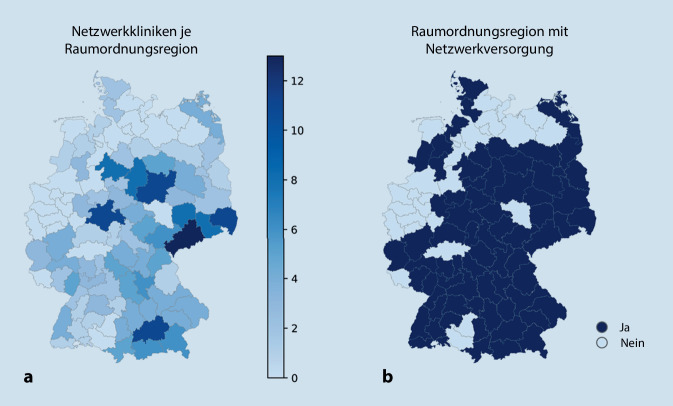

**Figure 2:**
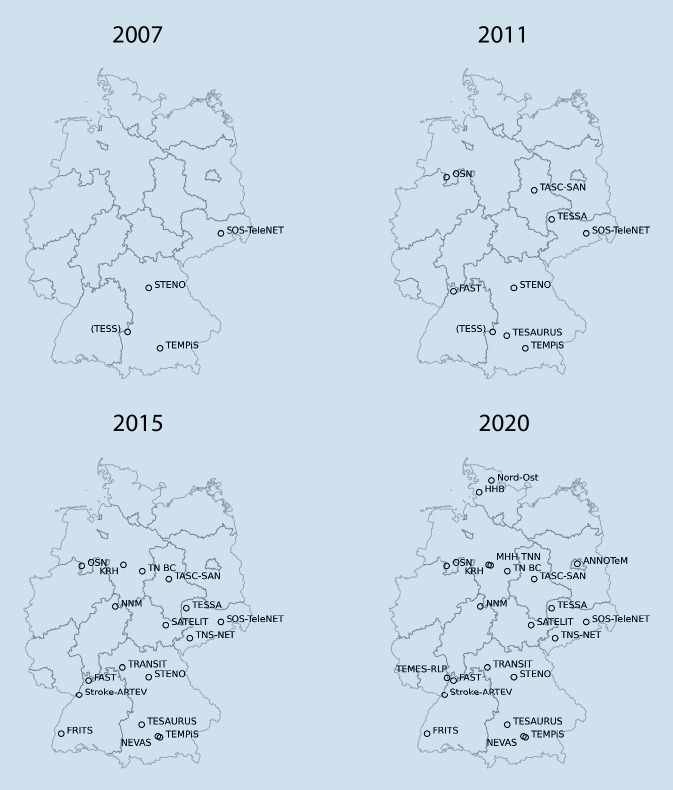

In Deutschland sind aktuell 22 telemedizinische Schlaganfall-Netzwerke aktiv, welche insgesamt 43 Zentren (pro Netzwerk: Median 1,5, IQA 1–3) sowie 225 Kooperationskliniken (pro Netzwerk: Median 9, IQA 4–17), davon 173 internistische (pro Netzwerk: Median 7,5, IQA 3–11) und 52 neurologische (pro Netzwerk: Median 0,5, IQA 0–5) Kliniken umfassen. Die geografische Abdeckung ist in Abb. 1 dargestellt. Das erste Netzwerk in Deutschland wurde im Jahre 2002 etabliert. Im Durchschnitt sind die Netzwerke seit 7,5 (±3,9) Jahren aktiv. Die zeitliche Etablierung ist in Abb. 2 dargestellt.

**Table 1:** 

Netzwerk	Zentren	Bundesland	Startjahr
ANNOTeM – Akutneurologische Versorgung in Nordostdeutschland mit telemedizinischer Unterstützung	Charité – Universitätsmedizin Berlin, Unfallkrankenhaus Berlin, Universitätsmedizin Greifswald	Brandenburg, Mecklenburg-Vorpommern, Sachsen-Anhalt	2017
FAST – Schlaganfallkonsortium Rhein Neckar	Universitätsklinikum Heidelberg	Baden-Württemberg, Hessen, Rheinland-Pfalz	2011
FRITS	Universität Freiburg	Baden-Württemberg	2014
HHB – Netzwerk Heide/Husum/Brunsbüttel	Westküstenkliniken Heide	Schleswig-Holstein	2016
NNM – NeuroNetz Mitte	Klinikum Kassel	Hessen, Nordrhein-Westfalen, Thüringen	2015
KRH – Netzwerk Klinikum Region Hannover	Klinikum Region Hannover	Niedersachsen	2015
MHH TNN – MHH Teleneurologie-Netzwerk	Medizinische Hochschule Hannover	Niedersachsen	2016
NEVAS – Neurovaskuläres Netzwerk Südwestbayern^a^	Klinikum der LMU München-Großhadern, BKH Günzburg, Klinikum Ingolstadt	Bayern	2013
OSN – Netzwerk Klinikum Osnabrück	Klinikum Osnabrück	Niedersachsen	2011
SATELIT – Schlaganfall Telemedizin Netzwerk in Thüringen	Universitätsklinikum Jena, Klinikum Altenburger Land	Thüringen, Sachsen-Anhalt, Sachsen	2013
SOS-TeleNET – Telemedizinische Schlaganfallversorgung in Ostsachsen Netzwerk	Universitätsklinikum Dresden	Sachsen	2007
STENO – Schlaganfallnetzwerk mit Telemedizin in Nordbayern	Universitätsklinikum Erlangen, Klinikum Nürnberg Universitätsklinik der Paracelsus Medizinischen Privatuniversität, Klinikum Bayreuth GmbH Klinik Hohe Warte	Bayern, Thüringen	2007
Stroke-ARTEV – Akute Regionale Telemedizinische Versorgungskette Karlsruhe	Städtisches Klinikum Karlsruhe	Baden-Württemberg, Rheinland-Pfalz	2012
TASC-SAN – Telemedical Acute Stroke Care Sachsen-Anhalt	Universitätsklinikum Magdeburg	Sachsen-Anhalt	2008
Telestroke Netzwerk Nord-Ost	Helios Kliniken Schleswig, Stralsund, Uelzen	Schleswig-Holstein, Mecklenburg-Vorpommern, Niedersachsen	2019
TEMES-RLP – Telemedizinisches Schlaganfallnetzwerk Rheinland-Pfalz	Klinikum Ludwigshafen, Universitätsmedizin Mainz, Westpfalzklinikum Kaiserslautern, Klinikum Idar-Oberstein, Kath. Klinikum Koblenz, Brüderhaus Trier	Rheinland-Pfalz	2016
TEMPiS – Telemedizinisches Schlaganfallnetzwerk Südostbayern	München Klinik Harlaching, medBo Regensburg (Kooperationsklinik der Universität Regensburg)	Bayern	2003
TESAURUS – Telemedizin & Schlaganfallversorgung Augsburger Region & Südwest-Bayern	Universitätsklinikum Augsburg	Bayern	2010
TESSA – Telemedizinisches Schlaganfallnetzwerk Nordwestsachsen	Klinikum St. Georg Leipzig, FKH Hubertusburg Wermsdorf	Sachsen	2010
TN BC – Teleneurologisches Netzwerk Braunschweig-Celle	Städt. Klinikum Braunschweig, AKH Celle	Niedersachsen	2015
TNS-NET – Teleneuromedizinisches Schlaganfallnetzwerk Südwestsachsen	Heinrich-Braun-Klinikum Zwickau, Klinikum Chemnitz, Helios Klinikum Aue	Sachsen	2012
TRANSIT – Transregionales Netzwerk für Schlaganfallintervention mit Telemedizin	Universitätsklinikum Würzburg, Neurologische Klinik Bad Neustadt, Leopoldina KH Schweinfurt	Bayern, Baden-Württemberg	2014

^a^NEVAS: Integration von TESS 2013

In jedem Netzwerk verfügt mindestens ein Zentrum über den Status einer nach den Richtlinien der Deutschen Schlaganfall-Gesellschaft (DSG) zertifizierten „überregionalen Stroke-Unit“. Von den internistischen Kooperationskliniken sind 30/173 (17,3 %) als „telemedizinisch vernetzte Stroke-Unit“ entweder durch die DSG oder nach Landesverfahren zertifiziert. Die Zertifizierungen in den neurologischen Kooperationskliniken umfassen eine (2 %) telemedizinisch vernetzte, 37 (71 %) regionale und 12 (23 %) überregionale Stroke-Units.

**Figure 3:**
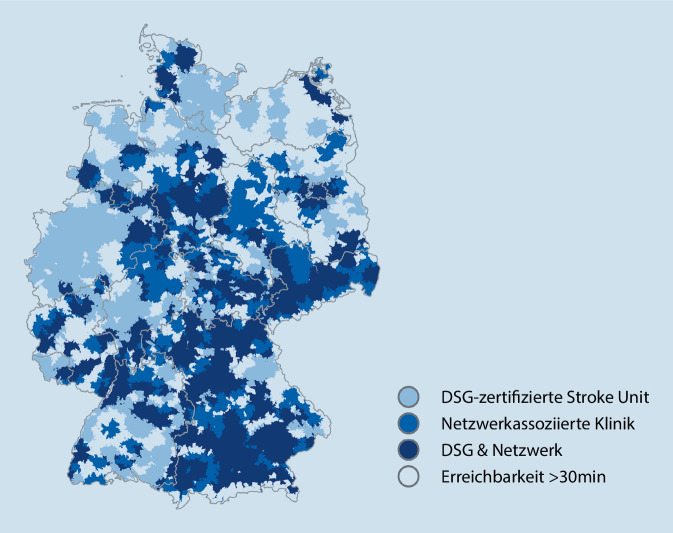

Die wohnortnahe Versorgung, definiert als Erreichbarkeit einer Schlaganfallstation innerhalb von 30 min Fahrtzeit, sowohl durch Netzwerkkliniken als auch durch DSG-zertifizierte Stroke-Units, ist grafisch in Abb. 3 dargestellt. Darauf basierend ergibt sich für 77 Mio. Menschen in Deutschland ein unmittelbarer Zugang zu einer wohnortnahen Schlaganfallversorgung, wobei Kliniken innerhalb von Schlaganfall-Netzwerken an der Abdeckung für 48 Mio. Menschen teilhaben. Eine zeitlich verzögerte Versorgung gemäß dieser Definition betrifft ungefähr 3 Mio. Menschen in Deutschland.

### Organisation

Alle Netzwerke arbeiten als integrative Netzwerke nach dem Hub-and-spoke-Prinzip, welches eine über die rein telemedizinische Beratungsfunktion hinausgehende Betreuung regionaler Kliniken umfasst. Die Indikationen für die Initiierung eines Telekonsils sind bei 21/22 (95,5 %) Netzwerken auf die Verdachtsdiagnose Schlaganfall fokussiert, bei 6/22 (27,3 %) im therapeutischen Zeitfenster, in aller Regel bis zu 24 h nach Symptombeginn. Ein Netzwerk untersucht explizit die telemedizinische Versorgung eines breiteren Spektrums akutneurologischer Erkrankungen [10].

**Figure 4:**
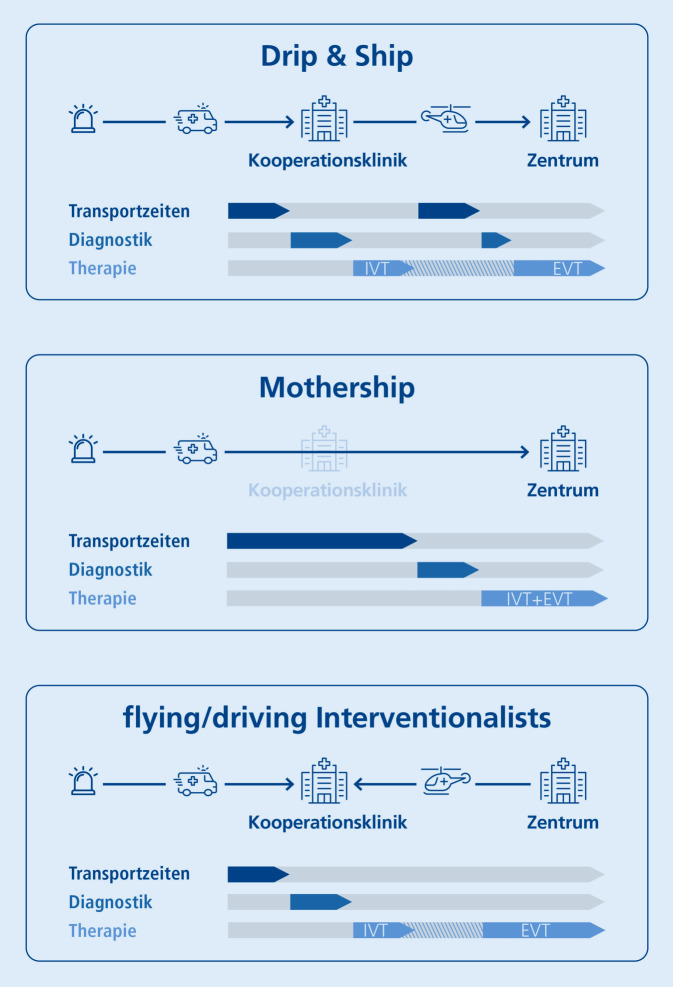

Eine Verlegung erfolgt primär nach dem Drip&Ship-Prinzip, wobei in 3 Netzwerken zusätzlich das Mothership- sowie in 2 Netzwerken zusätzlich das Flying/Driving-intervention-team-Prinzip praktiziert wird (Abb. 4).

Durchschnittlich stehen in den Zentren pro Netzwerk 2,5 (Median, IQA 1,5–4) zusätzliche Personalstellen zur Verfügung, wobei hierunter ärztliches, pflegerisches, therapeutisches und administrativ/wissenschaftliches Personal fällt. In allen Netzwerken steht die Telekonsiltätigkeit 24 h am Tag 365 Tage im Jahr zur Verfügung und wird von einem Arzt mit neurologischem Facharztstandard als Mindestqualifikation durchgeführt.

### Leistungszahlen

Im Jahr 2018 wurden 38.211 Telekonsile (pro Netzwerk: Median 1340, IQA 319–2758) durchgeführt. Diese Angabe basiert auf 21/22 (95 %) Netzwerken, da eines erst im Jahr 2019 etabliert wurde. Bei 71,1 % (27.174/38.211) der Patienten handelte es sich um einen Schlaganfall (G45, I61, I63). Bei einer Schlaganfallinzidenz von ca. 292/100.000 ist von einer telemedizinischen Mitbehandlungsrate von ca. 11 % auszugehen [11].

Die Thrombolyserate betrug 14,1 % (95 %-Konfidenzintervall 13,6–14,7 %) (basierend auf Daten von 17.389 Schlaganfallpatienten aus 17 Netzwerken). Eine Verlegung in ein netzwerkassoziiertes Zentrum zur Thrombektomie wurde bei 7,9 % (95 %-Konfidenzintervall 7,5–8,4 %) der Patienten initiiert (1182/14.875).

### Netzwerkarbeit und Qualitätssicherung

Die Netzwerkarbeit zentriert sich um regelmäßig stattfindende Audits (Median 1,5/Jahr, IQA 0,3–2) in den Kooperationskliniken vor Ort, die in 16/22 (72,7 %) Netzwerken primär die Überprüfung von Struktur- und Prozessqualität, in 14/22 (63,6 %) die Besprechung von Qualitätszahlen und in 6/22 (27,3 %) Schulungen beinhalten. Gemeinsame Visiten in den Kooperationskliniken führen 8 (36,4 %) Netzwerke durch. Multiprofessionelle Fortbildungen erfolgen mehrfach pro Jahr (Median 8, IQA 4–14), die ärztliche (Median 3, IQA 2–4), pflegerische (Median 4, IQA 1–6) und therapeutische (Median 2, IQA 0–4) Fortbildungen umfassen, die sowohl in den Zentren als auch in den Kooperationskliniken stattfinden. Regelmäßige Netzwerktreffen werden in 18 (81,8 %) Netzwerken durchgeführt (Median 1/Jahr, IQA 0,5–2). Dreizehn (59,1 %) der Netzwerke geben eine Kooperation mit dem Rettungsdienst an, welche vor allem Schulungen und die Etablierung eines Verlegungsmanagements beinhalten.

In allen Netzwerken erfolgt eine Qualitätssicherungsmaßnahme, entweder ausschließlich bei den telemedizinisch vorgestellten (11/22, 50 %) oder bei allen Schlaganfallpatienten, die in den Kooperationskliniken behandelt werden (11/22, 50 %). In den meisten Netzwerken (16/22, 72,7 %) erfolgt die Auswertung einmal jährlich. Insgesamt zeigt sich ein heterogenes Bild mit teils verpflichtenden Landesverfahren, teils freiwilliger oder durch das Netzwerk selbst durchgeführter Qualitätssicherung, jeweils in enger Anlehnung an die Qualitätsindikatoren der Arbeitsgemeinschaft deutschsprachiger Schlaganfall Register (ADSR).

### Finanzierung

Neun (40,9 %) Netzwerke geben eine Finanzierung durch die Krankenkassen an. Bei den Netzwerken in Sachsen und Thüringen (4/22, 18,2 %) werden sowohl die Zentren als auch die Kooperationskliniken über einen Rahmenvertrag fallbezogen pro Telekonsil durch die Krankenkassen finanziert. Fünf (22,7 %) Netzwerke, die in Bayern ansässig sind, erhalten von den Krankenkassen ein Zusatzentgelt für jeden behandelten Schlaganfallpatienten. Zwei (9,1 %) Netzwerke gaben eine Finanzierung über Drittmittel an. Für die übrigen 11 (50 %) Netzwerke existiert kein dezidiertes Vergütungssystem für die Zentren. Hier erfolgt eine Kompensation über die Kooperationskliniken, die pauschal oder fallbezogen für die Zentrumsleistungen aufkommen.

Die Kooperationskliniken erhalten in 17 (77,2 %) Netzwerken zumindest teilweise eine Refinanzierung für den Mehraufwand der telemedizinischen Schlaganfallversorgung über die Abrechnung der „Komplexbehandlung Schlaganfall mit Anwendung eines Telekonsildienstes“ (OPS [Operationen- und Prozedurenschlüssel] 8–98b), welche nur den Mehraufwand in der konsilempfangenen Klinik abbildet und nicht die Zentrumsleistung. Keine der Kooperationskliniken, die zur Finanzierung der Zentren Zahlungen leisten muss, bekommt diese erstattet.

Investitionen, die vor allem die technische Ausstattung umfassen, werden in 13 (59 %) Netzwerken als Eigenleistung getätigt, in 8 (36,4 %) durch Fördergelder auf Landesebene und in einem (4,5 %) Netzwerk durch Drittmittel.

## Diskussion

Die Ergebnisse dieser ersten bundesweiten Analyse zeigen, dass ein bedeutender Anteil der deutschen Bevölkerung heute schon Zugang zu einer Versorgung in Schlaganfall-Netzwerken hat und Schlaganfallpatienten auch in unterversorgten Regionen eine zeitgemäße, evidenzbasierte Akuttherapie angeboten werden kann. Allerdings bestehen auf struktureller Ebene noch uneinheitliche Standards in der Finanzierung und der Qualitätssicherung.

Seit 2002 tragen die Netzwerke dazu bei, evidenzbasierte Schlaganfalltherapien möglichst allen Patienten unabhängig von ihrem Wohnort zugänglich zu machen, mit positivem Effekt auf Qualitätsindikatoren in der Schlaganfallbehandlung sowie auf Sterblichkeit und Behinderung [1, 4–6, 12]. Mittlerweile wird die teleneurologische Schlaganfallbehandlung in Deutschland in 225 Kliniken eingesetzt, damit trägt sie zur Sicherstellung einer Schlaganfallversorgung auch in den ländlichen Gebieten bei. Derzeit wird etwa jeder 10. Schlaganfallpatient in Deutschland unter Inanspruchnahme der telemedizinischen Schlaganfallversorgung behandelt.

Auch auf geografischer Ebene lässt sich der Mehrwert der Netzwerke anhand der Einzugs- und Versorgungsgebiete eindrücklich darstellen. Vor allem im Süden und Osten des Bundesgebietes besteht eine hohe telemedizinische Versorgungsdichte (Abb. 1 und 3). In einzelnen nordöstlichen und westlichen Regionen zeigt sich unter Berücksichtigung von Netzwerkkliniken und netzwerkunabhängigen zertifizierten Stroke-Units eine Unterversorgung, sodass in diesen Gebieten die Implementierung einer verbesserten Netzwerkabdeckung geprüft werden sollte. Um bestehende Versorgungslücken zu schließen, bedarf es einer genauen Analyse geografischer und struktureller Voraussetzungen, die auch die Anforderungen an internistische Kooperationskliniken umfassen. Durch die Europäische Schlaganfallgesellschaft (European Stroke Organisation, ESO) wurden kürzlich Empfehlungen zur Umsetzung telemedizinscher Schlaganfallbehandlung in Form einer Richtlinie veröffentlicht, die sehr detailliert auf die Auswahl und Struktur telemedizinischer Kooperationskliniken eingeht [13]. Daran angelehnt sollte die Etablierung internistisch geführter telemedizinisch vernetzter Stroke-Units primär Regionen vorbehalten sein, in denen eine neurologische Unterversorgung herrscht. Eine Empfehlung zur Umsetzung in Deutschland ist durch die Kommission Telemedizinische Schlaganfallversorgung in Vorbereitung. Zur Erreichung definierter Ziele in der Versorgung von Schlaganfallpatienten für das Jahr 2030 ist letztlich unabdingbar, eine Kombination von etablierten neurologisch geführten zertifizierten Stroke-Units und telemedizinisch vernetzten Kliniken vorzuhalten [14].

Der relativ hohe Anteil an Telekonsilen, die keine Schlaganfälle darstellten (29 %), umfasst einerseits Differenzialdiagnosen zum Schlaganfall (sog. „stroke mimics“), die aufgrund der diagnostischen Unsicherheit in den Kooperationskliniken unter Schlaganfallverdacht vorgestellt wurden. Er zeigt aber auch den hohen Bedarf für eine breitere akutneurologische Beratung unter anderem auch bei unklarem klinischen Status auf [15]. Dieser Aspekt ist insbesondere vor dem Hintergrund der fehlenden neurologischen Expertise in dem Großteil der Kooperationskliniken ohne eigenständige neurologische Abteilung relevant, sodass eine inhaltliche Ausweitung des Telekonsildienstes wichtig scheint und aktuell in einem der Netzwerke wissenschaftlich evaluiert wird [10].

Untersuchungen zeigen, dass die Anwendung der zeitkritischen systemischen Lysetherapie bei Schlaganfallpatienten nicht in einem optimalen Bereich liegt und durch entsprechende fachliche Expertise und einen flächendeckenden Zugang verbessert werden kann [16–18]. Schon früh hat sich der Schlaganfall mit seinen gut audiovisuell erfassbaren klinischen Ausfällen zu einer Beispielanwendung für die Telemedizin etabliert und die Lysetherapie konnte sicher und effektiv in peripheren Krankenhäusern angewendet werden [4]. Eine Lyserate von über 14 % in den analysierten Kooperationskliniken liegt im Bereich der aktuellen Literatur (16,4 %) und kann als erfolgreiche Übertragung neurologischer Expertise mittels Telemedizin gewertet werden [5, 16, 19].

Seit 2015 steht mit der Thrombektomie für ausgewählte Patienten eine weitere hocheffektive Therapie des akuten ischämischen Schlaganfalls zur Verfügung [20], seit 2018 in einem Zeitfenster von bis zu 24 h nach Symptombeginn [21, 22]. Aufgrund hoher Strukturvoraussetzungen und Qualitätsansprüche steht die Thrombektomie nur in ausgewiesenen Zentren rund um die Uhr zur Verfügung. Möglichst allen Patienten den Zugang zur Thrombektomie gewährleisten zu können, setzt eine bestmögliche Patientenselektion und ein optimiertes Verlegungsmanagement innerhalb von Netzwerkstrukturen voraus [6, 8]. Man geht davon aus, dass ca. 10 % aller Patienten mit ischämischem Schlaganfall für eine Thrombektomie infrage kommen [23], sodass unsere Daten (8 % Verlegungen zur Thrombektomie) eine gute Patientenselektion über eine telemedizinische Beurteilung für diese hocheffektive Standardtherapie aufzeigen. Aktuelle Behandlungsraten der Thrombektomie liegen bei 6,5 % [16, 19], wobei die tatsächliche Behandlungsrate in unserer Population methodenbedingt nicht angegeben werden kann. Ein Zusammenhang der Rate an rekanalisierenden Therapien mit dem Urbanisierungsgrad einer Region unterstreicht die logistischen Schwierigkeiten der Umsetzung leitliniengerechter Therapien [19]. Die Anwendung verschiedener Konzepte über das primär angewandte Drip&Ship-Prinzip hinaus, die noch weiter wissenschaftlich beleuchtet werden müssen, und die zu intensivierende Zusammenarbeit mit dem Rettungsdienst im Hinblick auf eine optimale Transportlogistik stellen aktuelle und zukünftige Aufgaben der Netzwerke dar [24, 25].

Neben der rein quantitativen Darstellung des Versorgungsanteils der Netzwerke spielt die Erfassung der Qualität in den meist primär nichtneurologischen, sondern internistischen Kliniken mit telemedizinischer Betreuung eine wesentliche Rolle. Schon frühzeitig konnte die Verbesserung von Qualitätsindikatoren in der Schlaganfallbehandlung (z. B. Door-to-needle-Zeit, Dysphagiescreening, frühzeitige Physiotherapie) sowie des Outcomes nach 3 Monaten in telemedizinisch angeschlossenen Kliniken im Vergleich zu nichtbetreuten internistischen Kliniken aufgezeigt werden [26], welche auf andere Netzwerke übertragbar ist [6]. Die Implementierung fachlicher Standards stellt eine Voraussetzung für die Erfüllung der hohen Qualitätsansprüche in der Schlaganfallbehandlung dar. Anhand unserer Daten sind die erheblichen Aufwendungen ersichtlich, die unternommen werden, um dies gewährleisten zu können und spiegeln die Notwendigkeit einer adäquaten Ressourcenbereitstellung wider. Die Netzwerkarbeit umfasst letztlich nicht nur die Bereitstellung von Telekonsilen, sondern ermöglicht durch entsprechende Schulungsmaßnahmen den Aufbau von Schlaganfallfachkompetenz vor Ort.

Der teils heterogenen Bereitstellung qualitätsbezogener Leistungszahlen liegt die fehlende Vereinheitlichung von Qualitätssicherungsmaßnahmen zugrunde. Der Schlaganfall unterliegt keiner gesetzlich geforderten Qualitätssicherung, auf Landesebene gibt es unterschiedliche Anforderungen, in Einzelfällen auch auf Konzernebene. Es besteht die Verpflichtung zur Qualitätssicherung für nach den Kriterien der DSG zertifizierte Stroke-Units. Hieraus ergibt sich die Problematik, dass mitunter einzelne Kliniken unterschiedlichen Bestimmungen unterliegen und eine netzwerkübergreifende Qualitätssicherung erschwert wird. Hier würde ein bundeseinheitliches Verfahren die Vergleichbarkeit der Qualitätsindikatoren vereinfachen und eine Optimierung der Schlaganfallversorgung ermöglichen. Geeignete Qualitätsindikatoren sollten neben den Prozesszeiten in der Notaufnahme (Door-to-imaging-Zeit, Door-to-needle-Zeit) auch weiterführende Stroke-Unit-spezifische Indikatoren, wie Schluckscreening und zeitnahe Initiierung medizinischer Therapien, umfassen, da sich dadurch die erfolgreiche Implementierung von Standards in den Kooperationskliniken gut widerspiegelt.

Eine wesentliche Aufgabe der Zentren ist die Unterstützung von Zertifizierungsbestrebungen der telemedizinisch vernetzten Stroke-Units, welche mit 17 % bislang nur ein geringer Anteil der internistischen Kooperationskliniken umgesetzt hat. Durch die Zertifizierungskriterien der DSG werden Anforderungen an telemedizinische Kooperationskliniken sehr gut definiert. Problematisch ist, dass einige Qualitätsanforderungen, die im OPS-Code „Neurologische Komplexbehandlung des akuten Schlaganfalls mit Anwendung eines Telekonsildienstes“ abgebildet sind, teilweise nicht flächendeckend erfüllt werden können, wie z. B. die tägliche Anwesenheit eines Neurologen [27]. Hier kommen insbesondere geografische Gegebenheiten zum Tragen, sodass der Fachärztemangel in ländlichen Regionen, dem die Etablierung der telemedizinischen Netzwerke ursprünglich zugrunde liegt, in weiterer Folge die Finanzierung dieser Struktur behindert.

Aktuell ist die Finanzierung der telemedizinischen Schlaganfallversorgung sehr uneinheitlich gestaltet. Teils ist eine nachhaltige Finanzierung wie z. B. in Bayern gewährleistet, in anderen Bundesländern finanzieren die Kooperationskliniken die Zentrumsleistung ohne entsprechende Refinanzierung, was durch die Nichtabbildung der Zentrumsleistung im OPS begründet ist. Konkret wird gefordert, dass die Finanzierungslücke der zentralen Netzwerkleistungen, welche die Kosten für die telemedizinischen Leistungen und deren Vorhaltung, die Organisation und Qualitätssicherung sowie die technische Ausstattung und Instandhaltung umfassen, in einem neuen bundeseinheitlichen Finanzierungsmodell berücksichtigt wird. Mögliche Umsetzungen wären z. B. eine Einbeziehung in die bestehende DRG(„diagnosis related groups“)-Systematik, eine Kompensation über Zusatzentgelte oder über Zentrumszuschläge.

Die vorgelegte Arbeit berichtet erstmals umfassende Daten aller aktiven telemedizinischen Schlaganfall-Netzwerke in Deutschland. Limitierend ist die unvollständige Darstellung qualitätsbezogener Leistungszahlen. Ebenso besteht kein Einblick, wie strukturiert im Einzelfall die Datenakquise erfolgte. Dies unterstreicht jedoch die Forderung nach einheitlichen Vorgaben zur Qualitätssicherung in der Schlaganfallbehandlung. Trotz umfassender Recherche kann nicht ausgeschlossen werden, dass einzelne kleinere Netzwerkverbunde der Erhebung entgangen sind, was jedoch quantitativ von untergeordneter Größenordnung wäre. Insgesamt konnte anhand unserer Umfragestudie der Grundstein für eine netzwerkübergreifende Abstimmung von Strukturmerkmalen, Behandlungsstandards und die einheitliche Erhebung von Qualitätssicherungsdaten gelegt werden, um eine kontinuierliche Qualitätsverbesserung in der flächendeckenden Schlaganfallversorgung zu gewährleisten. Als etablierte Anwendung dienen telemedizinische Schlaganfall-Netzwerke auch als Vorbild für andere medizinische Bereiche, die eine spezialisierte Versorgung auf hohem Niveau bisher nicht flächendeckend anbieten konnten.

## Fazit für die Praxis

- Telemedizinische Schlaganfall-Netzwerke leisten einen wesentlichen Beitrag zur flächendeckenden und wohnortnahen Schlaganfallversorgung.
- Telemedizinische Schlaganfall-Netzwerke tragen zur Umsetzung leitliniengerechter Therapieempfehlungen bei.
- Eine netzwerkübergreifende Abstimmung von Strukturmerkmalen und Behandlungsstandards kann zur flächendeckenden Qualitätssteigerung führen.
- Eine Vereinheitlichung von Qualitätssicherungsmaßnahmen ist anzustreben.
- Enormer personeller Aufwand erfordert eine Sicherstellung der Finanzierung und entsprechender Ressourcen.

## Supplementary Information


