# Supplementary material for: Telemedicine in stroke—pertinent to stroke care in Germany
Source: Nervenarzt. 2021 May 27;92(6):593–601. [Article in German] doi: 10.1007/s00115-021-01137-6 (PMC8184549; doi:10.1007/s00115-021-01137-6)
Supplement: Supplementary file 1 [file 115_2021_1137_MOESM1_ESM.pdf]

Telestroke-Netzwerke Bestandsaufnahme

Netzwerk	
Ärztlicher Leiter/Koordinator	
Klinikum	
Telefonnummer	
E-Mail-Adresse	
Startjahr	

Basis

1. Anzahl Zentren

Zentrum 1: ☐ überregionale SU ☐ regionale SU ☐ EVT

Zentrum 2: ☐ überregionale SU ☐ regionale SU ☐ EVT

Zentrum 3: ☐ überregionale SU ☐ regionale SU ☐ EVT

Zentrum 4: ☐ überregionale SU ☐ regionale SU ☐ EVT

Zentrum 5: ☐ überregionale SU ☐ regionale SU ☐ EVT

Zentrum 6: ☐ überregionale SU ☐ regionale SU ☐ EVT

- Möglichkeit weitere Zentren einzugeben -

2. Bundesland/-länder

☐ länderübergreifend

Einzugsgebiet: qkm

Einwohner (im Einzugsgebiet/Kreis der telemedizinisch angeschlossenen Kliniken, ohne Zentren)

Flächendeckung im Einzugsgebiet ☐ ja ☒ nein

3. Kooperationskliniken

Anzahl ☐

Bitte Details zu den einzelnen Kliniken in die zusätzliche Tabelle einpflegen.

Anzahl weiterer Kliniken im Einzugsgebiet, die nicht Teil des Netzwerks sind:
☐ internist., ☐ neurolog., mit EVT-Kapazität: ☐

4. Netzwerk-Zertifizierung vorhanden

☐ keine

☐ Neurovaskuläres Netzwerk nach DSG ☐ Netzwerkzertifizierung nach DIN ISO

☐ Sonstiges ☐

Konsile - Leistungszahlen

5. Verpflichtende Indikationen für Telekonsil (laut Vereinbarung/Vertrag)

☐

☐ Fokus auf Schlaganfall

☐ nur Pat. mit zeitkritischer Therapieoption (def. über Zeitfenster) vs. ☐ jeder Schlaganfall (ohne zeitliche Begrenzung)

☐ weitere Indikationen außer Schlaganfall (laut Vereinbarung/Vertrag), bitte angeben:
☐

☐ Neurologische Anfragen außerhalb der o.g. Indikationen werden dennoch bearbeitet; bitte konkretisieren: ☐

6. Behandelte Pat. in den Kooperationskliniken und Konsile gesamt

Behandelte Pat. gesamt, n	2017	2018
Ischäm. Schlaganfall, n		
TIA, n		
Hirnblutungen, n		

	2017	2018
Anzahl Telekonsile, n		
Schlaganfalldiagnosen, n (%)[1]		
- Ischäm. Schlaganfall, n (%)[2]		
- System. Lysen, n (%)[3]		
- TIA, n (%)[2]		
- Hirnblutungen, n (%)[2]		
Andere Diagnosen, n (%)[1]		
Verlegungen, n (%)[1]		
Verlegungen zur EVT, n (%)[1]		

Prozentangaben beziehen sich auf: [1] Telekonsile, [2] Schlaganfalldiagnosen, [3] Ischäm. SA

7. Behandelte Schlaganfallpatienten in den Zentren (Zahlen 2018)

Zentrum (analog zu Pkt.1):	1	2	3	4	5	6	gesamt
Schlaganfalldiagnosen, n							
- Ischäm.Schlaganfall, n (%)							
- System. Lysen, n (%)							
- TIA, n (%)							
- Hirnblutungen, n (%)							

Möglichkeit weitere Zentren einzugeben

8. Leistungszahlen Endovaskuläre Therapie (2018)

Leistungszahlen EVT vorhanden? [ ] ja [x] nein

Teilnahme an QS für EVT? [ ] ja [x] nein wenn ja, welche: ______

Anzahl EVTs in den Zentren gesamt: ______; davon verlegt aus dem Netzwerk, n (%) ______

Anzahl Patienten aus Kooperationskliniken, die eine EVT erhielten, n (%), (bezogen auf alle ischämischen Schlaganfälle in den Koop.kliniken): ______

Leistungszahlen zu Verlegungszeiten/Transportlogistik vorhanden [ ] ja [x] nein

EVT-Konzept: [ ] drip&ship [ ] mothership [ ] flying intervention team [ ] Sonstiges: ______

9. Wissenschaftliche Aktivität

[ ] keine wissenschaftliche Aktivität

[ ] regelhafte begleitende wissenschaftliche Auswertung, durch (z.B: Institut) ______

[ ] Effizienzanalyse erfolgt/geplant: ______

[ ] laufende prospektive wissenschaftliche Studie: ______

[ ] Datenbank zur Nutzung für gezielte Fragestellungen vorhanden

Koordination/Netzwerkarbeit

10. Mitarbeiter aller Zentren

Bitte als Stellenanteil (z.B.1,0 VK) angeben, der für das Netzwerk tätig ist bzw. über Netzwerk finanziert wird

Anzahl gesamt ______ VK □ Ärztl. Leiter/in ______ □ weitere ärztl. MA ______

□ wissenschaftl. MA ______ □ Geschäftsstelle ______ □ Pflegeexperte/in ______

□ Therapeut/in ______ □ medizin. Dokumentationsassistent/in ______ □ sonstige ______

11. Dienstsystem des Telekonsildienstes

Telekonsildienst 24/7 [x] ja [ ] nein, ______

Durch [ ] Assistenzarzt [ ] Facharztstandard [ ] Facharzt [ ] Oberarzt

Stellenanteil: Regelarbeitszeit ☐ VK, exklusiv für Telekonsildienst ☐ ja ☒ nein

Dienstzeit ☐ VK, exklusiv für Telekonsildienst ☐ ja ☒ nein

☐ Sonstiges: ______

Wenn mehr als 1 Zentrum, Rotation des Konsildienstes? ☐ ja ☒ nein

Einbindung Neuroradiologie ☒ Telekonsildienst ☐ jederzeit kontaktierbar

☐ sonstiges: ______

Einbindung Neurochirurgie ☒ Telekonsildienst ☐ jederzeit kontaktierbar ☐ Erstellung eines eigenen Konsils ☐ sonstiges: ______

12. Netzwerkarbeit

Audits vor Ort: Frequenz ______

Inhalt: ☐ Überprüfung Standards/Struktur/Prozesse ☐ Präsentation Qualitätszahlen

☐ Sonstiges: ______

Gemeinsame Visiten in der Koop.klinik? ☐ ja ☒ nein

Fortbildungen ärztlich: im Zentrum ______ x/Jahr, vor Ort ______ x/Jahr

Fortbildungen Pflege: im Zentrum ______ x/Jahr, vor Ort ______ x/Jahr

Fortbildungen Physio/Logo/Ergo: im Zentrum ______ x/Jahr, vor Ort ______ x/Jahr

Netzwerktreffen ☐ ja ☒ nein wenn ja, wie oft: ______

Kooperation mit dem Rettungsdienst (z.B. Teilnahme an Netzwerktreffen, Konzept für EVT-Verlegungen, Schulungen des RD, Auswertung Verlegungszeiten etc) ☐ ja ☒ nein

Wenn ja, bitte konkretisieren: ______

Standards/SOPs

13. SOP-Entwicklung

Welche SOPs/Leitlinien werden konkret benutzt?

☒ SOPs/Leitlinien der Fachgesellschaften; welche (DGN, AHA/ASA, ESO): ______

☐ Es wurden eigene SOPs entwickelt/die obigen Leitlinien wurden übernommen und angepasst

☐ Keine spezifischen, verschriftlichten Leitlinien. Die Empfehlungen richten sich nach der besten klinischen Praxis.

Werden die Partnerkliniken in die Leitlinienauswahl/-erstellung einbezogen? ☐ ja ☐ nein

14. Spezifische SOPs

SOPs vorhanden für:

Akuttherapie ischämischer Schlaganfall: ☒ ja ☐ nein

Systemische Thrombolyse ☒ ja ☐ nein

Mechanische Rekanalisation ☒ ja ☐ nein

Sekundärprävention ischämischer Schlaganfall: ☒ ja ☐ nein

Akuttherapie hämorrhagischer Schlaganfall: ☒ ja ☐ nein

Weitere SOPs zerebrovaskuläre Krankheiten:

Weitere SOPs andere neurologische Krankheiten:

Wie erfolgt das Verlegungsmanagement? ☐ standardisierte Reihenfolge/SOP

☒ ad hoc-Auswahl durch Dienstarzt

15. Umsetzung aktueller Studienergebnisse

Wird in Ihrem Netzwerk Folgendes empfohlen/durchgeführt?

Sekundärprävention nach CHANCE/POINT: ☒ ja ☐ nein

Erweitertes Thrombektomiefenster nach DAWN/DEFUSE: ☐ ja, wird regelmäßig durchgeführt ☒ prinzipiell ja, aber Perfusionssoftware für CT/MRT steht meist nicht zur Verfügung ☐ nein, wird nicht empfohlen

Systemische Lysetherapie nach WAKE-UP/EXTEND-Kriterien: ☐ ja, wird regelmäßig durchgeführt ☒ prinzipiell ja, aber MRT/ Perfusionssoftware für CT steht meist nicht zur Verfügung ☐ nein, wird nicht empfohlen

Interventioneller PFO-Verschluss bei Pat. <60J mit kryptogenem Schlaganfall und mindestens mittelgroßem Shuntvolumen/Nachweis Vorhofseptumaneurysma: ☒ ja ☐ nein

16. Qualitätssicherung im Netzwerk

Wird durchgeführt ☒ ja ☐ nein

wenn ja, ☒ bei allen telemedizinisch vorgestellten Pat. ☐ bei allen Schlaganfallpat.

☒jährlich ☐andere Frequenz ______

☐Stichprobe, bitte beschreiben: ______

☒Papierbögen ☐elektronisch, welche Software: ______

Verantwortlich für Dateneingabe: ☒ Zentrum ☐ Koop.klinik

Kliniken nehmen teil an regulärer verpflichtender QS ☒ja ☐nein wenn ja,welche: ______

Wenn nein, welche Parameter werden erfasst: ☒analog zu ADSR ☐andere: ______

______

Finanzierung

17. Finanzierung des/der Zentrums/Zentren

☐Krankenkasse (z.B. Rahmenvertrag), ☐fallbezogen, ______€

☐Landeszuschuss ☐fallbezogen, ______€

☐Finanzierung durch Umlage

☐Kooperationsklinik bezahlt Abschlag/Gebühr: , ______€

☐Sonstiges, ______

18. Finanzierung der Kooperationsklinik

☒OPS 8.98b

Anzahl an internistischen Kooperationskliniken, die die Voraussetzungen für die OPS 8.98b grundsätzlich erfüllen: ______

Anzahl an Patienten bei denen die OPS 8.98b kodiert wird, n (%) (bezogen auf alle Schlaganfallpatienten in den Kooperationskliniken 2018): ______

☐Krankenkasse (z.B. Rahmenvertrag), ☐fallbezogen, ______€

☐Sonstiges, ______

19. Investitionen

Hardware, Software sowohl im Zentrum als auch in den Kooperationskliniken

☒Förderung, durch ______ ☐Eigenleistung der Klinik

☐Sonstiges, ______

Technik

20. Systeme

MEYTEC: ☐Videokonferenz ☐Bildübertragung ☐Konsilerstellung

Andere: ☐Videokonferenz ☐Bildübertragung ☐Konsilerstellung

☐Videokonferenz ☐Bildübertragung ☐Konsilerstellung

☐Videokonferenz ☐Bildübertragung ☐Konsilerstellung

Bei anderen Anbietern als MEYTEC bitte Kontaktdaten angeben:

21. Technische Ausstattung

Internetanbindung/Bandbreite Zentrum: Mbit/s* Kliniken: Mbit/s* (* wenn bekannt Up- & Downstream)

Nutzung ☐der Krankenhausleitung ☒gesonderte Leitung

Angaben zu Hardware: PC

Laptop

Letzte Erneuerung der Hardware (Jahr):

Betriebssystem:

Transfer-Modul MEYTEC in Verwendung ☐ja ☒nein

Bildgebung in PACS der Zentren übertragbar ☐ja ☒nein

Technische Lösung für Qualitätssicherung ☐ja ☒nein wenn ja, welche:

22. Schriftliches Konsil

☐Word-Dokument ☒ generiertes pdf

☐digital übertragbar ☐in KIS der Koop.klinik integrierbar

☐in KIS des Zentrums integrierbar (z.B. nach Verlegung)

Informationen werden standardisiert in einer Datenbank erfasst ☐ja ☒nein

Wenn ja, welche Software: ☐MEYTEC ☐Excel – Tabelle ☐Sonstiges:
